# Harris' Medical and Dental Dictionary. Fourth Edition

**Published:** 1878-01

**Authors:** 


### BIBLIOGRAPHICAL.

---

*Harris' Dictionary of Dental Surgery.*—Fourth Edition.

We have received from the Publishers, Messrs. Lindsay and Blakiston, a copy of the new edition of "Harris' Dictionary of Dental Surgery," just issued, or as the new work is styled, "A Dictionary of Medical Terminology, Dental Surgery and the Collateral Sciences." This volume is the result of a good many months' patient labor and research on the part of the editor, Prof. F. J. S. Gorgas.

No work could be less of a showy character than the revision of a dictionary, and no work could be imagined, unless it be statistics, more dry and unsavory. Yet the editor has with untiring patience and the closest scrutiny, scanned every word of every page of the previous edition of the dictionary, with the result of making a much more valuable book than its predecessor.

The work of revision has not consisted simply of elimination, though many obsolete words are erased; but many valuable amendments to definitions are made, and many definitions made to conform to the more modern phrases of belief, very many entirely new terms in medicine and dentistry are inserted, and a number of pages of new matter added.

The whole comprises a work almost as useful to the medical as to the dental student, while to the latter it is an indispensable necessity, being indeed the only one of the sort in existence.

A new and valuable feature is the description of all the articles of the materia medica employed in the practice of dentistry, and the dental uses added to others before referred to in this work.

The publishers have made this edition of the dictionary, in point of typography, a great improvement on the previous editions. The work is stereotyped, and the print more elegant.

